# Proteotranscriptomic Analysis Reveals Stage Specific Changes in the Molecular Landscape of Clear-Cell Renal Cell Carcinoma

**DOI:** 10.1371/journal.pone.0154074

**Published:** 2016-04-29

**Authors:** Benjamin A. Neely, Christopher E. Wilkins, Laura A. Marlow, Dariya Malyarenko, Yunee Kim, Alexandr Ignatchenko, Heather Sasinowska, Maciek Sasinowski, Julius O. Nyalwidhe, Thomas Kislinger, John A. Copland, Richard R. Drake

**Affiliations:** 1 Department of Cell and Molecular Pharmacology and Experimental Therapeutics, Medical University of South Carolina, Charleston, South Carolina, United States of America; 2 Department of Microbiology and Molecular Cell Biology, Eastern Virginia Medical School, Norfolk, Virginia, United States of America; 3 Department of Cancer Biology, Mayo Clinic Comprehensive Cancer Center, Jacksonville, Florida, United States of America; 4 Department of Radiology, University of Michigan Medical School, Ann Arbor, Michigan, United States of America; 5 Department of Medical Biophysics, University of Toronto, Toronto, Ontario, Canada; 6 Princess Margaret Cancer Center, Toronto, Ontario, Canada; 7 INCOGEN, Inc., Williamsburg, Virginia, United States of America; 8 Venebio Group, LLC, Richmond, Virginia, United States of America; 9 Leroy T. Canoles Jr. Cancer Research Center, Eastern Virginia Medical School, Norfolk, Virginia, United States of America; University of Texas Health Science Center, UNITED STATES

## Abstract

Renal cell carcinoma comprises 2 to 3% of malignancies in adults with the most prevalent subtype being clear-cell RCC (ccRCC). This type of cancer is well characterized at the genomic and transcriptomic level and is associated with a loss of *VHL* that results in stabilization of HIF1. The current study focused on evaluating ccRCC stage dependent changes at the proteome level to provide insight into the molecular pathogenesis of ccRCC progression. To accomplish this, label-free proteomics was used to characterize matched tumor and normal-adjacent tissues from 84 patients with stage I to IV ccRCC. Using pooled samples 1551 proteins were identified, of which 290 were differentially abundant, while 783 proteins were identified using individual samples, with 344 being differentially abundant. These 344 differentially abundant proteins were enriched in metabolic pathways and further examination revealed metabolic dysfunction consistent with the Warburg effect. Additionally, the protein data indicated activation of ESRRA and ESRRG, and HIF1A, as well as inhibition of FOXA1, MAPK1 and WISP2. A subset analysis of complementary gene expression array data on 47 pairs of these same tissues indicated similar upstream changes, such as increased HIF1A activation with stage, though ESRRA and ESRRG activation and FOXA1 inhibition were not predicted from the transcriptomic data. The activation of ESRRA and ESRRG implied that HIF2A may also be activated during later stages of ccRCC, which was confirmed in the transcriptional analysis. This combined analysis highlights the importance of HIF1A and HIF2A in developing the ccRCC molecular phenotype as well as the potential involvement of ESRRA and ESRRG in driving these changes. In addition, cofilin-1, profilin-1, nicotinamide N-methyltransferase, and fructose-bisphosphate aldolase A were identified as candidate markers of late stage ccRCC. Utilization of data collected from heterogeneous biological domains strengthened the findings from each domain, demonstrating the complementary nature of such an analysis. Together these results highlight the importance of the VHL/HIF1A/HIF2A axis and provide a foundation and therapeutic targets for future studies. (Data are available via ProteomeXchange with identifier PXD003271 and MassIVE with identifier MSV000079511.)

## Introduction

Renal cell carcinoma accounts for 90% of kidney cancers, and 2 to 3% of malignancies in adults [1]. In 2012, kidney cancer was the ninth and fourteenth most common cancer in men and women worldwide, respectively [2], predominantly affecting men 2:1 [1]. Of these worldwide cases in 2012, there was a 42% mortality rate [2], and in a survey of over 340,000 RCC cases between 2001 and 2010, 25% of cases were stage III or IV [3]. Within renal cell carcinoma, clear-cell renal cell carcinoma (ccRCC) is the most prevalent subtype comprising 70 to 80% of cases [1]. Detection of ccRCC is the result of an incidental diagnosis 50 to 60% of the time, and survival is greatly affected by tumor grade at diagnosis. The 5-year survival rate for grade I and II tumors is 88.9% and grade III and IV tumors is 65.6% and 41.7%, respectively [4]. For this reason it is imperative to develop a clear understanding of the molecular pathogenesis of ccRCC in order to identify new targets related to metastatic ccRCC.

The defining genetic characteristic of ccRCC is the loss of chromosome 3p and/or mutations of the von Hippel-Lindau tumor suppressor gene (*VHL*), especially in sporadic ccRCC where 92% have inactivated *VHL* by mutation, hypermethylation or deletion [5]. In a survey of data from The Cancer Genome Atlas (TCGA) encompassing 12 major cancer types, *VHL* mutation occurred in over 50% of ccRCC cases and was not a factor in other malignancies [6], although some studies have observed *VHL* mutation in over 80% of ccRCC cases [7]. With the loss of VHL, hypoxia-inducible factor 1α (HIF1A) and HIF-2α (HIF2A; also referred to as EPAS1) are stabilized [8, 9], each regulating at least 350 gene loci [10, 11]. Specifically, stabilization of HIF1A leads to downstream changes resulting in an oncologic shift in glucose and glutamine metabolism consistent with the Warburg effect (first described in the 1920s [12] and expertly reviewed in [13]). This results in increased glucose uptake and increased glycolytic flux, with the top-half of the glycolytic pathway facilitating intermediates for the pentose phosphate pathway and biosynthesis required for tumor growth. The majority of glucose goes to lactate fermentation, thereby uncoupling oxidative metabolism in the tricarboxylic acid (TCA) cycle [14]. In order to facilitate fatty acid synthesis, the TCA cycle instead relies on glutamine flux [15], with primary outputs being citrate and malate. Importantly, HIF1A and HIF2A stabilization have different effects on promoting tumor growth (reviewed in [16]): HIF1A activates glycolytic genes while HIF2A promotes growth and angiogenesis [17, 18]. Moreover, later stages of ccRCC have higher levels of HIF2A and HIF1A and HIF2A are thought to be antagonistic [9, 18].

The seminal TCGA study of ccRCC confirmed many Warburg effect-related changes at the genomic/transcript level and correlated changes with stage and survival [19]. This large-scale characterization study identified increased DNA methylation in higher stage tumors, as well as key transcriptional hubs (HIF1A/ARNT, MYC/MAX, SP1, FOXM1, JUN and FOS). It was also found that down-regulation of genes in the TCA cycle, and up-regulation of genes in the pentose phosphate pathway and fatty acid synthesis correlated with poor survival. Specifically, reduced transcript levels of AMP-activated kinase (AMPK) and increased levels of acetyl-CoA carboxylase (ACC) corresponded to worse survival, changes that would contribute to increased fatty acid synthesis. In addition to the TCGA work, other transcriptomic studies have been performed using tumor-normal paired samples, identifying almost 6000 differentially expressed genes in ccRCC, and 31 genes required for tumorigenesis have been confirmed *in vitro* [20]. Additionally, genes related to adipogenesis have been identified, likely explaining the hallmark lipoic clear cell morphology of ccRCC [21]. Together these studies have improved our understanding of the molecular changes at the gene and transcript level required for the ccRCC phenotype.

In addition to characterizing genomic and transcriptomic changes in ccRCC, there have been a series of shotgun-proteomic analyses [22–25]. In general these studies have confirmed dysregulated metabolic patterns supporting the Warburg shift predicted at the transcript level [22, 24], including increased abundance of proteins in the pentose phosphate pathway related to tumor aggressiveness [23]. Studying ccRCC across different biological domains (gene, transcript, protein, metabolite) is essential since changes are not linear between these domains. For example, the follow up proteomic analysis by Zhang *et al*. [26] to the TCGA study of colorectal cancer found a mean Spearman’s rank correlation coefficient between transcript and protein levels of 0.23. Similarly, a recent metabolomic study of ccRCC compared their results to the TCGA ccRCC study (albeit using different samples) and found little to no correlation between transcript and metabolite levels [27]. In another study, a follow-up metabolomic analyses of stage-dependent proteomics of ccRCC demonstrated protein abundance does not correspond to stage-dependent changes in metabolites [25, 28]. It is important to utilize the complementary nature of different techniques across domains in order to identify changes at each biological hierarchy (abundance, isoforms, post-translational modifications, *etc*.) that are driving phenotypic changes.

In the current study we utilized proteomic analysis to characterize the molecular landscape of ccRCC and interrogated changes in protein abundance and biological pathways with ccRCC stage. Moreover, we used a previously published transcriptomic data set from the same sample cohort to strengthen our conclusions. We also identified cofilin-1 (CFL1), profilin-1 (PFN1), nicotinamide N-methyltransferase (NNMT), and fructose-bisphosphate aldolase A (ALDOA), as candidate markers of late stage ccRCC. This combined proteotranscriptomic analysis not only strengthened our understanding of the underlying metabolic changes that occur in ccRCC, but also highlights probable changes in gene regulation that result in changes to the molecular phenotype of ccRCC. By utilizing data from heterogeneous biological domains, we have improved both analyses and provide the foundation for future studies of therapeutic targets.

## Methods

### Sample collection and storage

In January, 2000, Mayo Clinic began collecting and storing fresh-frozen patient matched normal renal and tumor tissue samples of individuals undergoing nephrectomy. The tissue samples are linked to the Nephrectomy Registry database and are also available to other investigators conducting renal cancer research. Collections of pathological specimens occurred in an IRB approved manner such that subjects could not be identified. Approximately 300 patients per year diagnosed with renal cell carcinoma undergo nephrectomy at the Mayo Clinic. Patients presenting with local tumors, locally advanced tumors, and patients with metastatic disease undergoing cytoreductive nephrectomy were candidates for this study. The gender breakdown for patients presenting with RCC is approximately 65% male and 35% female, and the average patient age was 65 years old. Deidentified patient matched normal and ccRCC tissues were collected under a Mayo Clinic Institutional Review Board approved protocol (IRB#1746–03 first approved September 2, 2003; “Cancer Research Investigations Using Discarded Tissues") to use tissue for molecular analyses for research purposes only (Mayo Clinic OHRP number: FWA00005001). Samples were collected from surgical resections, snap frozen in liquid nitrogen and stored at -80°C. A centralized pathology review on all tumor samples was performed to confirm tumor histologic classification, TNM stage and grade. The following definitions were used: stage I is localized ccRCC with tumor less than 7 cm; stage II is localized ccRCC with tumor >7 cm; stage III is invasive ccRCC into Gerota fascia; stage IV ccRCC is metastic disease to a distal organ. There were 177 tissue samples used in the current study taken from 84 patients comprised of 34 stage I, 40 stage II, 42 stage III, and 52 stage IV tumor normal-adjacent pairs as well as 9 corresponding metastasis tissues.

### Immunohistochemistry

A tissue microarray was created using paired normal-adjacent and tumor tissue cores from 54 patients with stage I ccRCC. The resulting formalin-fixed, paraffin-embedded block was cut into 5 μm sections, deparaffinized, hydrated, antigen retrieved and blocked with diluent that contained background reducing components (Dako; Carpinteria, CA). The section was probed for 6-phosphofructokinase (PFKP antibody at 1:1500; LifeSpan BioSciences, Inc.; Seattle, WA) and pyruvate kinase (PKM2 antibody at 1:2500; Cell Signaling Technology; Danvers, MA). The specificity of the antibodies was confirmed using positive and negative controls from breast tumor tissue for PFKP and lung normal and tumor tissue for PKM2. Positive controls were performed and carried out to high titers to demonstrate decreased staining intensity (S1 Fig). Negative controls were performed with the absence of primary antibody (S1 Fig). For detection, the Envision Dual Labeled Polymer kit (Dako) was used according to the manufacturer’s instructions and then lightly counterstained with Gill I hematoxylin (Sigma-Aldrich) before dehydration and mounting. Images were obtained at 20X using an Aperio AT2 Scanscope (Leica Biosystems, Buffalo Grove, IL). An average IHC staining intensity score for each core was generated with triplicate measurement using an in-house Imagescope algorithm. The number of cores read varied from 36 to 48, and the values were compared using a two-sample *t*-test (Excel).

### Sample preparation

Individual tissues (10 to 15 mg) were processed by trifluoroethanol (TFE) solubilization and sonication as adapted from Wang *et al*.[29]. Briefly, tissue in 50% TFE was disrupted sequentially by repeated probe sonication and heat/vortexing (1 hr, 60°C), with at least two repeats. At this step, 10 μg of an internal protein standard, maltose-binding periplasmic protein from *Escherichia coli*, was added to the lysate. Reducing agent [tris(2-carboxyethyl)phosphine] and alkylating agent (iodoacetamide; IAA) were added, followed by overnight trypsin digestion (1:50 based on protein concentration) at 37°C. The resulting peptides were desalted with a C-18 column (1cm x 1cm), dried down by SpeedVac and reconstituted with Mobile Phase A [5% ACN, 0.1% formic acid (FA), 0.005% heptafluorobutyric acid (HFBA)]. Pooled samples were composites of five samples, and these were tumor normal-adjacent matched between the pooled samples.

### Data acquisition

For the pooled samples, a fully automated five-cycle two-dimensional high-performance liquid chromatography sequence was set up as previously described [30]. Peptides were loaded onto a 7-cm Kasil fritted pre-column (150 μm inner diameter) packed with 3.5 cm of 5 μm Magic C-18 100 Å reversed-phase material (Michrom Bioresources Inc., Auburn, CA) followed by 3.5 cm of Luna 5 μm SCX 100-Å strong cation exchange resin (Phenomenex, Torrance, CA). Samples were loaded automatically from a 96-well microplate autosampler at 3 μl/min using the EASY-nLC system (Thermo Scientific). The pre-column was connected to a fused silica analytical column (8 cm long, 75 μm inner diameter) via a microsplitter tee (Thermo Scientific) to which a distal 2.0 kV spray voltage was applied. The analytical column was pulled to a fine electrospray emitter using a laser puller. For peptide separation on the analytical column, a water-ACN gradient, controlled by the EASY-nLC (Thermo Scientific), was applied at an effective flow rate of 400 nL/min. Ammonium acetate salt bumps (8 μl) at concentrations of 100, 150, 200, and 500 mM were sequentially loaded, and peptides were eluted by a water-ACN gradient as described previously [30]. Sample analysis was performed on an LTQ Orbitrap XL (Thermo Scientific) using previously described instrument parameters [30]. For individual tissue sample analysis, tumor-normal pairs were analyzed by tandem mass spectrometry temporally close together. Digests were resuspended in 20 μL Mobile Phase A and a 15 μL aliquot of this peptide solution was separated on a 12 cm x 0.075 mm fused silica capillary column packed with 5 μm diameter C-18 beads (The Nest Group, Southborough, MA) across a 90 min linear gradient from 5% ACN, 0.1% FA, 0.005% HFBA to 95% ACN, 0.1% FA, 0.005% HFBA at 300 nL/min. The LC was interfaced by electrospray ionization with an LTQ (ThermoFinnigan, San Jose, CA). Data-dependent analysis was used to perform MS/MS on the five most intense ions between *m/z* = 400 and 2000 in each MS spectra with a minimum signal of 1000 cps. Dynamic exclusion was used with a repeat count of two and an exclusion duration of 180 s.

### Data processing

Raw data from pooled sample tandem mass spectrometry runs were converted to mzXML files using ReAdW (v1.1) and searched against the Human UniProtKB SwissProt database (2011_3 release; 20,227 sequences plus the addition of *E*. *coli* maltose-binding periplasmic protein, malE, P0AEX9) using X!Tandem (CYCLONE v2011.12.01.1), OMSSA (v2.1.8), and MyriMatch (v2.1.97) search algorithms. The search was conducted with a fragment ion mass tolerance of 0.40 Da and a parent ion tolerance of 10 ppm. Complete tryptic digestion was assumed with one allowed missed cleavage site. Methionine oxidation was specified as a variable modification and alkylation of cysteine with IAA as a static modification. For protein inference minimization, an in-house grouping scheme was applied, reporting only proteins with substantial peptide information [31]. Target/decoy searches were performed to experimentally estimate the protein false discovery rate, which was determined to be <1%. Protein identifications with at least two unique tryptic peptides were considered [31]. The mass spectrometry data have been deposited to MassIVE(MSV000079511).

Raw data from individual sample tandem mass spectrometry runs were converted to peak list (mgf format) using MSConvert (ProteoWizard 3.0.4243; Jan 3, 2013). The default parameters were used, including ‘Prefer Vendor for Peak Picking’. These mgfs were searched with Mascot (v2.4.1; Matrix Sciences) using the following parameters: trypsin as the enzyme with a maximum of two mis-cleavages; 1+, 2+, and 3+ charged peptides; carbamidomethyl (C) as a fixed modification, and protein N-term acetylation, deamidation (NQ) and oxidation (M) as variable modifications; instrument type was ESI-TRAP; a precursor tolerance of 2 Da and fragment ion tolerance 0.5 Da. These thresholds were chosen based on a test analysis which resulted in a <0.1% local FDR (above identity threshold) while maintaining the highest number of protein hits. The database used was a *Homo sapiens* database (taxon ID: 9606) retrieved from the 2013_04 release of the UniProtKB SwissProt database along with the SwissProt varsplic database, a cRAP database (common Repository of Adventitious Proteins, v 2012.01.01; The Global Proteome Machine) and the entry *E*. *coli* malE (P0AEX9), resulting in 38,480 sequences. A separate search was also performed against a reversed decoy version of this database in order to calculate global FDR. Resulting.dat files were loaded into ProteoIQ (v2.3.08; NuSep, Inc; Bogart, GA) and protein results were filtered by requiring a 5% FDR and minimum of two peptides per protein identification. Following this step, there were 350,630 identified peptides (10,590 unique) belonging to 786 proteins. Three of these proteins were contaminants and malE, which were removed from the analysis resulting in 783 proteins identified experiment wide. Spectral counts were normalized according to total spectral counts per sample and exported from ProteoIQ for downstream analysis. The mass spectrometry proteomics data have been deposited to the ProteomeXchange Consortium [32] via the PRIDE partner repository with the dataset identifier PXD003271 and 10.6019/PXD003271.

Affymetrix Human Genome U133 Plus 2.0 array data (.CEL files) were retrieved from the NCBI Gene Expression Omnibus (GSE 53757), which contains data for the 144 arrays used by von Roemeling *et al*. [20]. Prior to analysis, a subset was created of just the 94 arrays (paired tumor normal-adjacent samples) that correspond directly to samples used in the current proteomic study. The data were processed with the *affy* R-package using RMA normalization. The array was annotated with the *hgu133plus2*.*db*, *annotate*, and *R2HTML* R-packages. A key linking sample IDs and mzIdentML files between this proteomic analysis and the transcriptomic analysis can be found in S1 Table.

### Data analysis

Proteomic data from the pooled samples were evaluated by calculating adjusted spectral counts (asp), as recently described [33]. The asp values for each protein were used to perform a Wilcoxon rank sum test using the exact method (Matlab, v8.5.0.197613; MathWorks) followed by a Benjamini-Hochberg (BH) procedure to correct for multiple hypothesis testing. Proteins were considered differentially abundant between tumor and normal-adjacent samples at BH adjusted *p* < 0.05. Normalized protein spectral count data from patient matched tumor and normal-adjacent samples were used to calculate a tumor to normal-adjacent ratio for each protein, which was log_2_ transformed. These log_2_ transformed ratios were evaluated with a moderated *t*-test (*limma* package [34]; R v3.2.1) followed by a BH procedure to correct for multiple hypothesis testing. This comparison was performed within tumor stages or by using data from all stages. Proteins were considered differentially abundant at a BH adjusted *p* < 0.05. These results were analyzed through QIAGEN’s Ingenuity® Pathway Analysis (IPA®, QIAGEN, Redwood City) by specifying human as a species, experimentally observed confidence, and not limiting the search space to a tissue or cell line. All other parameters were defaults within IPA. Pathway enrichment was performed within IPA using a Fisher's exact test (right-tailed), and pathways were considered significant at BH adjusted *p* < 0.05, and at least two proteins per pathway. Results were evaluated within stages and across all stages. Upstream regulator analysis was performed in IPA which uses a z-score algorithm, such that an activation z-score ≥ 2 is considered activated and ≤ -2 is considered inhibited. Regulator effects were predicted using the regulator effects algorithm in IPA that links upstream regulator analysis (upstream regulators) and downstream effects analysis (disease or functions) and merges networks with overlapping targets. Array data were also evaluated in IPA by using RMA normalized values and a moderated *t*-test. Probes with an absolute log_2_ fold-change ≥ 2 and BH adjusted *p* < 0.001 were used for IPA analysis (n = 1003) using the same parameters as the proteomic IPA analysis. These transcriptomic results were compared to identify overlapping patterns of pathway enrichment and/or upstream regulator effects. These same parameters were used to evaluate the following comparable published proteomic data sets: Perroud *et al*.[25] Supp. Table 3 (180 proteins), Masui *et al*.[35] Supp. Table 5 (29 proteins), White *et al*.[22] Supp. Table 7 (55 proteins), and Zhao *et al*.[24] Online resource 1 (213 proteins).

## Results

### Molecular phenotype of ccRCC

**Fig 1:**
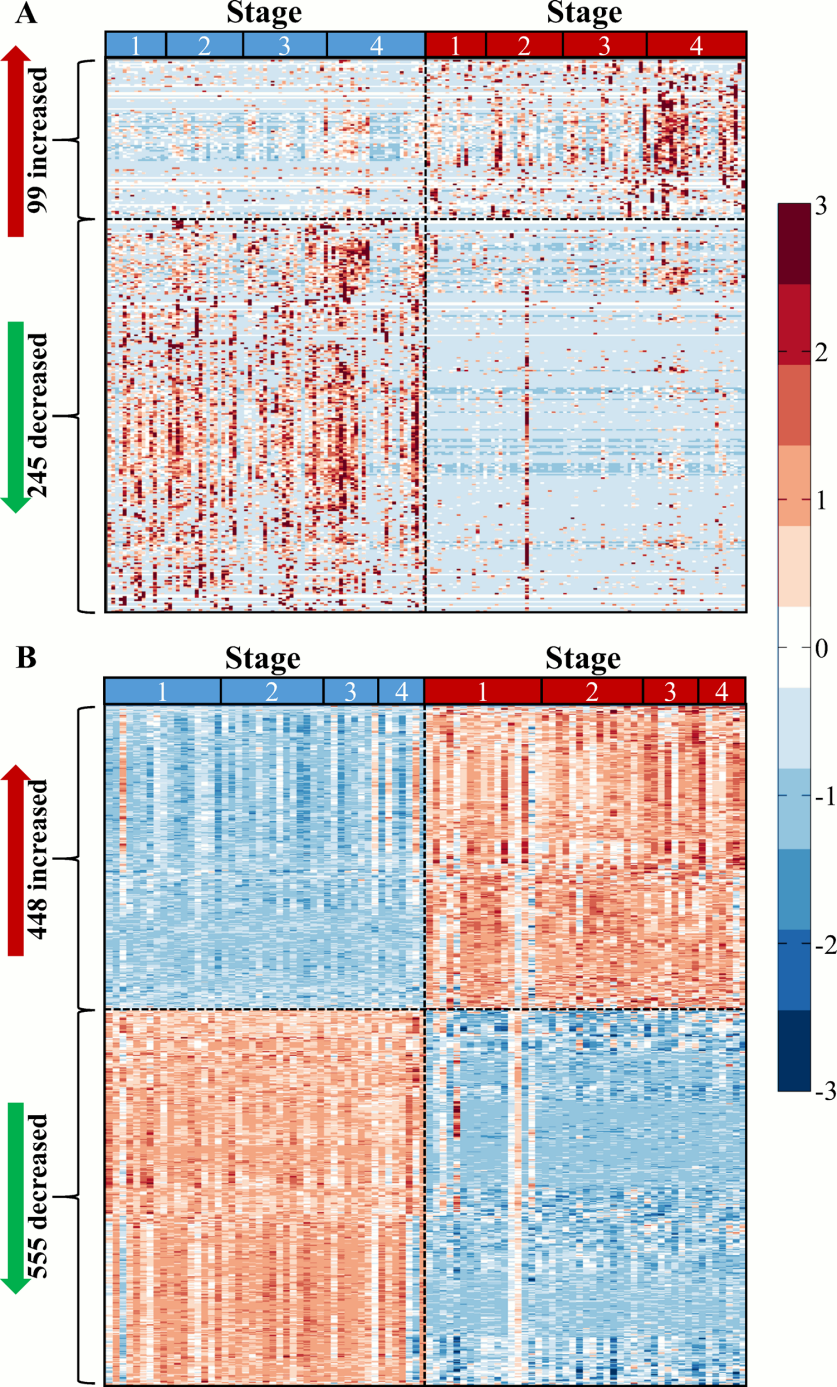
Differential protein abundance and gene expression between tumor and normal-adjacent ccRCC tissues. (A) Heatmap of 344 proteins with differential abundance between tumor and normal-adjacent samples (moderated *t*-test, Benjamini-Hockberg adjusted *p*-value < 0.05). (B) Heatmap of 1003 genes with differential expression between 94 tumor and normal-adjacent samples (moderated *t*-test BH adjusted *p* < 0.001 and absolute fold-change ≥ 4). Scale bar is standard deviation units around the mean of each protein abundance or gene expression level.

In order to characterize proteomic changes related to ccRCC, two proteomic approaches were employed. The first approach was to catalog proteins in ccRCC tissue samples by using in-line multi-dimensional separation techniques prior to high resolution bottom-up shotgun proteomic analysis [30]. This approach was used on pooled samples from tumor or normal-adjacent tissues: four stage I tumor, two stage I normal-adjacent, two stage II tumor, two stage II normal-adjacent, two stage III tumor, two stage III normal-adjacent, two stage IV tumor and two stage IV normal-adjacent. The average Pearson's linear correlation coefficient (*r*) within replicates was 0.8835 demonstrating low intra-condition variability (S2 Fig). From these pooled samples, 1551 proteins were identified (S2 Table), and by comparing label-free quantification between tumor and normal-adjacent tissues, 290 were detected as differentially abundant at BH adjusted *p* < 0.05 (Wilcoxon rank sum test). Of these, 249 (85.9%) were decreased and 41 (14.1%) were increased in tumor samples. To further characterize the molecular changes involved in the progression of ccRCC, samples from tumor and normal-adjacent tissues from 84 individuals with stage I (17 pairs), II (20 pairs), III (21 pairs), and IV (26 pairs) ccRCC were evaluated individually using a shorter separation gradient than the pooled analysis. Nine individuals with stage IV ccRCC had samples from metastasized tissue in addition to tumor and normal-adjacent tissues. Using this approach, 783 proteins were identified (global protein FDR < 5%) and normalized spectral counts were calculated (S3 Table). In order to normalize data across the individual analysis data set, each patient’s normal-adjacent tissue was used as a reference to calculate fold-change values for each protein in the tumor tissue (or metastasis tissue). These values were log-transformed and 344 proteins were identified as being differentially abundant (moderated *t*-test, BH adjusted *p* < 0.05; Fig 1A). Similar to the pooled analysis, 245 (71.2%) were decreased, and 99 (28.2%) were increased in tumor samples.

Initially the pooled and individual analysis approaches were compared by evaluating overlap of identified proteins. To directly compare the two datasets, seven isoforms were removed from the individual analysis to eliminate ambiguity in assignments, leaving 776 proteins. Of the proteins identified in the individual analysis, 663 (85.4%) were identified in the pooled analysis (S3A Fig). Next, the fold-change of each of these shared proteins in each analysis was visualized using a scatter plot (S3C Fig), and 540 (81.5%) of the proteins exhibited conserved directionality change, showing good agreement between the analyses. Then the 290 proteins that were differentially abundant in the pooled analysis were compared to the 342 proteins that were differentially abundant in the individual analysis (S3B Fig). There was considerable overlap between the analyses (169 proteins), with 58.3% and 49.4% of the differentially abundant proteins in the pooled analysis or individual analysis being shared, respectively. Overall this showed good agreement between analyses, and the individual analysis was used for further bioinformatic interrogation.

Since a subset of these tissues had previously been analyzed using gene expression arrays [20], we were interested in identifying parsimony between the analytical domains. Previously 144 samples were analyzed, and 94 samples (47 individuals) of these were directly related to the 84 individuals used in the current proteomic study. Therefore a subset analysis comparing tumor and normal-adjacent tissues was performed with just data from these 94 arrays. Of the 54,677 probes present, 37,334 had a BH adjusted *p* < 0.05 (moderated *t*-test; S4 Fig). Similar to von Roemeling *et al*. [20], we applied an additional fold-change cutoff to identify a subset of highly differentially expressed genes. Whereas von Roemeling applied a cutoff of log_2_ fold-change of 1 (resulting in 5937 genes), in the current comparison a log_2_ fold-change cutoff of 2 was used to identify 1003 differentially expressed genes between tumor and normal-adjacent tissues (Fig 1B). Genes with increased and decreased expression were approximately evenly distributed (S4 Fig).

**Fig 2:**
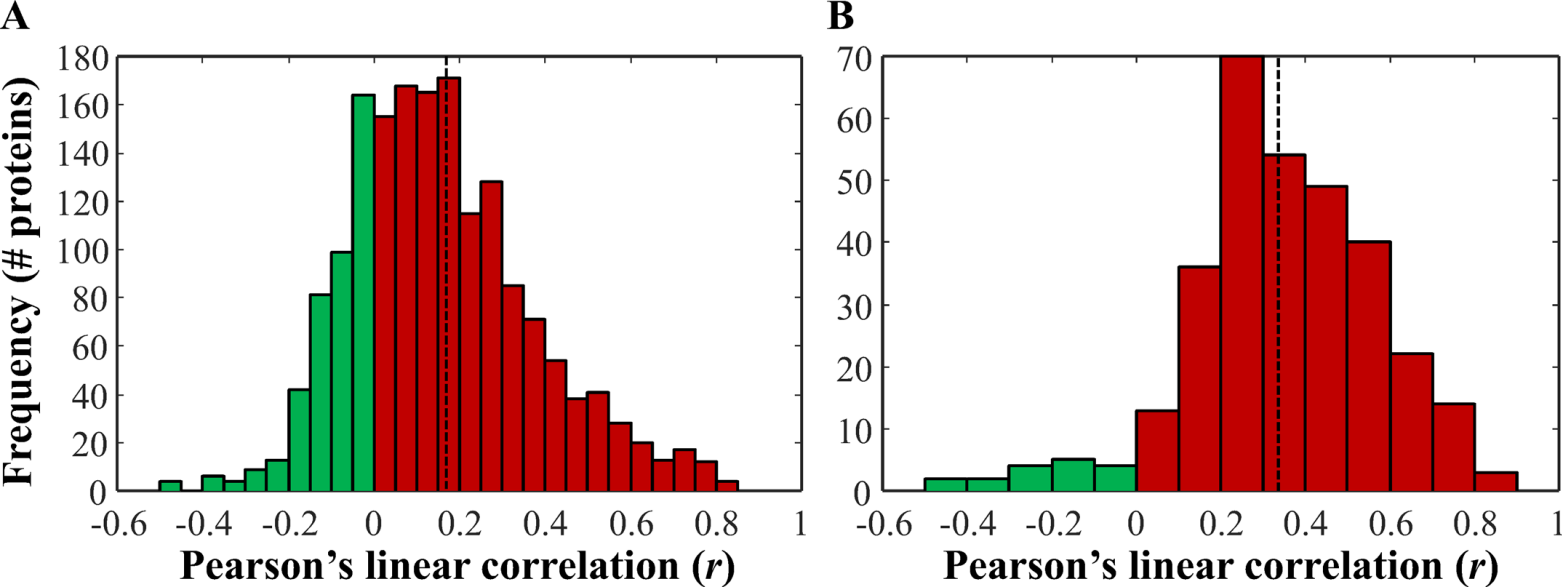
Correlation of differentially abundant proteins and respective gene expression levels in matched samples. (A) There were 725 proteins identified which had 1764 corresponding probes in the corresponding transcriptomic data. Pearson's linear correlation coefficient was used to correlate normalized spectral count levels and RMA normalized microarray data in matched samples (94 samples). The average Pearson's linear correlation coefficient (*r*) was 0.157 (dotted line). (B) Distribution of *r* for just the 344 differentially abundant proteins, of which 318 were also measured in the corresponding transcriptomic study. The average *r* was 0.347 (dotted line).

After performing a subset analysis on the gene expression data to identify differentially expressed genes, we evaluated the correlation between protein abundance and gene transcript levels. Of the 783 proteins identified, there were 770 unique HGNC gene symbols, of which 725 overlapped with 1764 gene probes. The majority of these were positively correlated (1285; 72.8%), and the average Pearson's linear correlation coefficient (*r*) was 0.157 (Fig 2A). When only proteins that were differentially abundant between tumor and normal-adjacent tissue were interrogated (344, of which 318 had complementary expression data), the majority were positively correlated (301; 94.7%) with only 17 negatively correlated, and an average *r* of 0.347 (Fig 2B). Interestingly, 287 of the 318 probes that corresponded to differentially abundant proteins were also differentially expressed (BH adjusted *p* < 0.05). This provided evidence that important changes at the protein level were largely a reflection of signal at the transcript level, although the relatively low average correlation indicates this relationship was not linear.

### Pathway enrichment analysis

**Fig 3:**
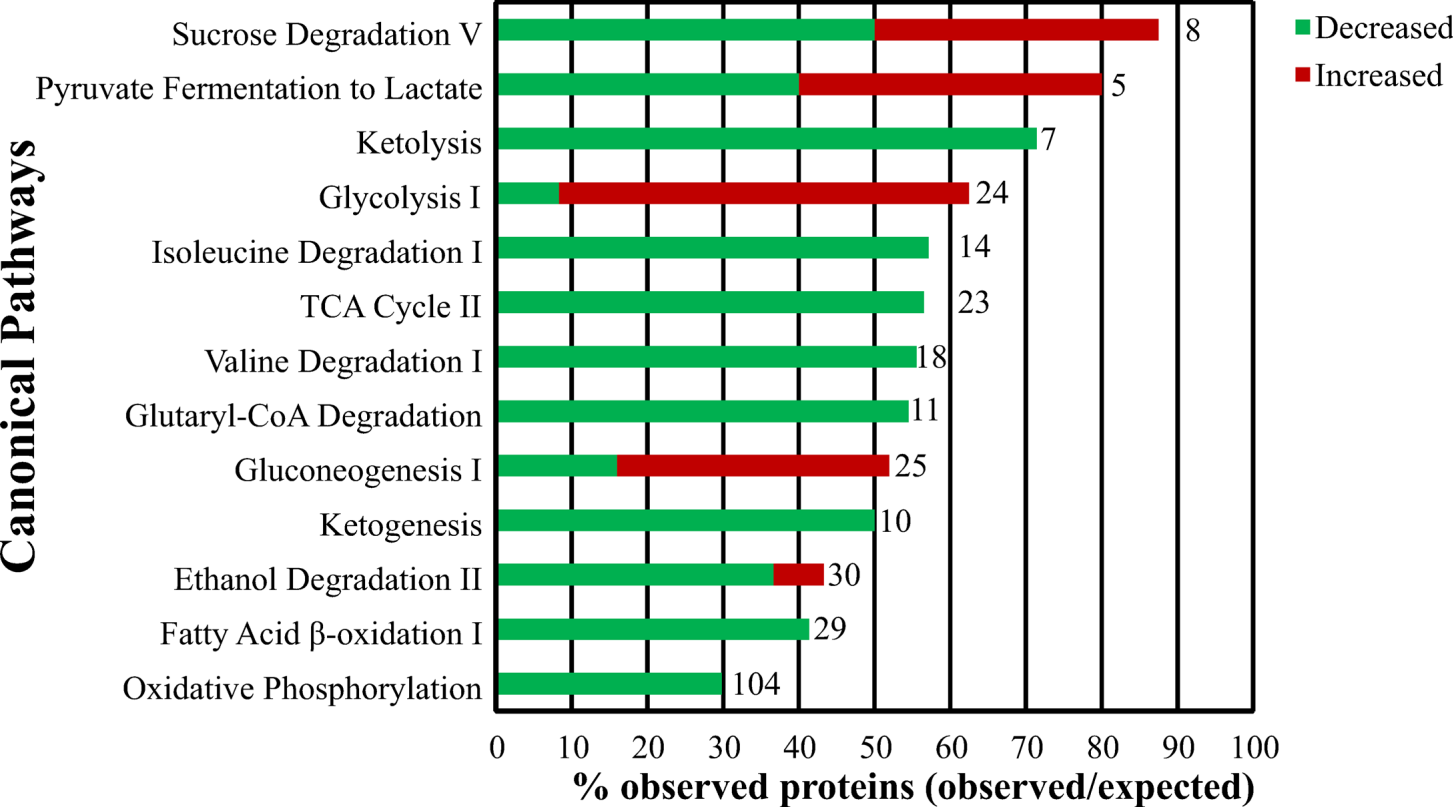
Enriched pathways related to metabolic dysfunction in ccRCC. Pathway enrichment analysis using IPA was performed using the 344 proteins with differential abundance between normal and tumor samples. Of the 88 pathways identified at an FDR < 5% and containing more than one protein, the following top 13 pathways are shown based on their relationship to Warburg effect related changes. The ratio of enrichment (or % observed) is further divided into those proteins increased or decreased in tumor samples. The total number of possible (or expected) proteins in each pathway is given to the right of the bar.

Differentially abundant proteins identified in the analysis of individual samples were used to identify 88 canonical pathways that were enriched in ccRCC. The top enriched pathways were those related to metabolism (Fig 3). There was decreased abundance of proteins involved in ketolysis, the TCA cycle, ketogenesis, fatty acid β-oxidation, oxidative phosphorylation and degradation of isoleucine, valine, glutaryl-CoA, and ethanol, while glycolysis and gluconeogenesis had increased protein abundance. Pyruvate fermentation to lactate via LDH was more complicated with LDHA and LDHAL6B being increased in tumor tissues (2.55 and 0.69 log_2_ fold-change, respectively), while LDHB and LDHAL6A were decreased (-2.11 and -0.24 log_2_ fold-change, respectively). Likewise, sucrose degradation had increased levels of ALDOA, ALDOC, and TPI1 (0.90, 0.27, and 0.61 log_2_ fold-change, respectively), and decreased levels of ALDOB, KHK, and TKFC (-3.36, -0.74, and -0.90 log_2_ fold-change, respectively; TKFC was previously listed as DAK in the UniProtKB entry Q3LXA3 in S3 and S4 Tables, and was modified to TKFC in August 2015 after this analysis was completed). Also, although all the TCA cycle proteins identified were significantly decreased, the degree of change varied. Specifically, proteins responsible for transforming citrate and malate to *cis*-aconitate and oxaloacetate, respectively, were the most decreased proteins in the TCA cycle (ACO2–1.9 log_2_ fold-change and MDH2–1.6 log_2_ fold-change, respectively), supporting accumulation of these two substrates for fatty acid biosynthesis (similar to [22]).

**Fig 4:**
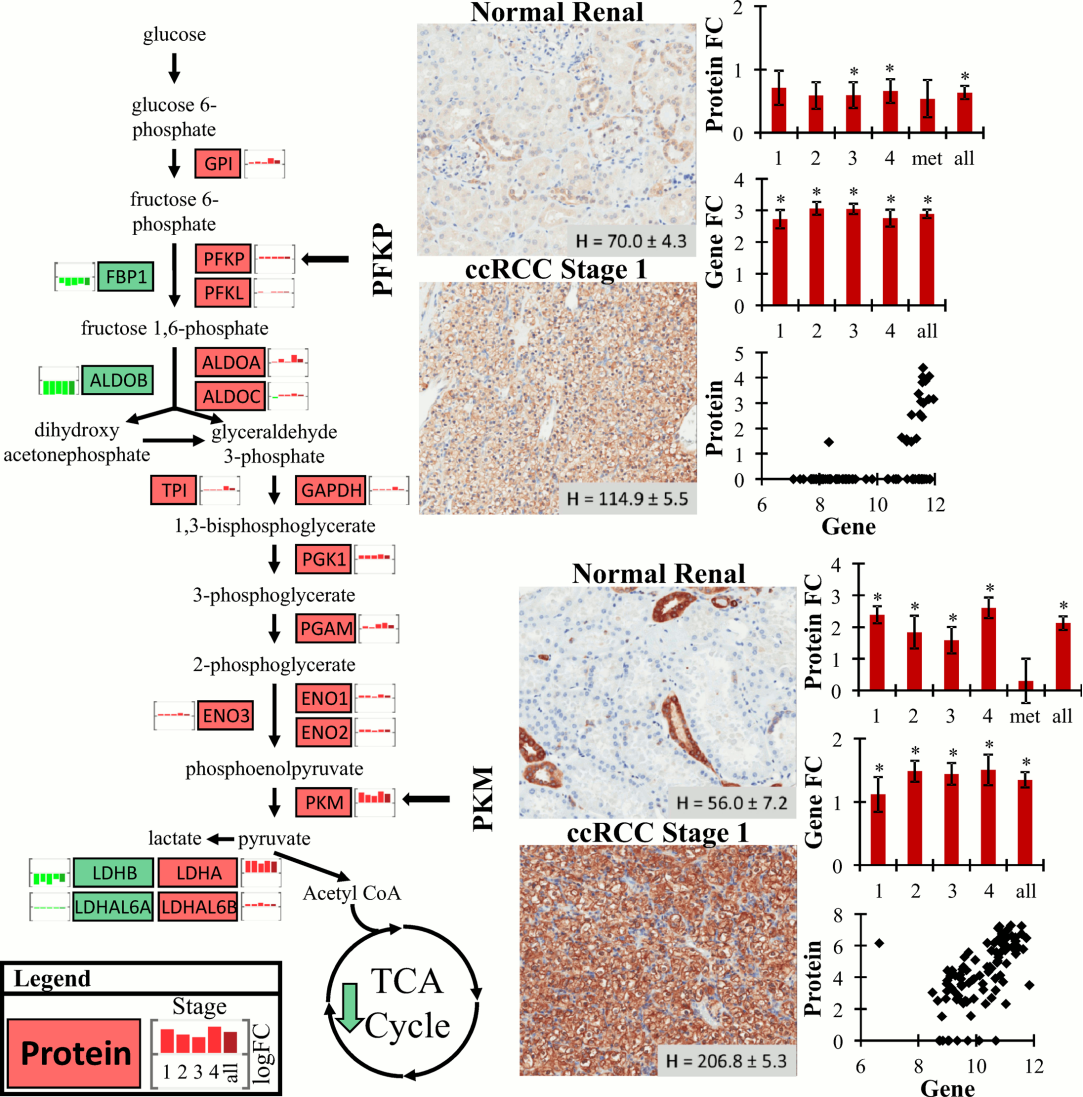
Protein abundance changes with stage in the glycolysis pathway. Using only proteins that were differentially abundant at each stage, the glycolysis pathway was interrogated. Directionality of change is indicated by red (increased in tumor) or green (decreased in tumor) and the small bar graph next to each protein symbol in the pathway is protein fold-change (tumor/normal) at stage I, II, III, IV and then all stages (from left to right). Levels of two proteins, PFKP and PKM, were confirmed using IHC staining of stage I ccRCC tissue (other stages were not evaluated by IHC). A representative IHC image is shown for PFKP and PKM along with average staining intensity (H value) ± standard error of a stage I ccRCC TMA. For both PFKP and PKM the average log_2_ fold-change (FC) levels ± standard error for protein abundance (stage I-IV and metastasis tissue) and gene expression (stage I-IV) are displayed as bar graphs (‘*’ indicates significance, BH adjusted *p* < 0.05). Below each pair of bar graphs is a scatter plot of log_2_ normalized spectral counts (protein) versus RMA normalized array gene expression data from the 94 tissues with proteomic and transcriptomic data.

To better understand the metabolic dysregulation in ccRCC, we evaluated protein abundance changes in glycolytic proteins (Fig 4). Contrary to previous metabolomic studies indicating the top-half of glycolysis being asynchronously increased in ccRCC [27], at the protein level there were widespread increases in glycolytic proteins. Many of these proteins were increased by relatively the same fold-change at all stages (similar to [25, 28]), although some proteins were highest at stage IV (GPI, ALDOA, ALDOC, TPI, GAPDH, PGK1, PGAM, ENO1, ENO3 and PKM). Using IHC, increased levels of PFKP and PKM in stage I ccRCC were confirmed. Using a TMA of stage I ccRCC tumor and normal-adjacent tissue, PFKP was increased 1.64-fold in tumor tissue (*t*-test, *p* < 0.001), and increased 3.63-fold in tumor (*t*-test, *p* < 0.001). Expectedly, the protein immunohistochemistry levels aligned with protein abundance changes, but not transcript expression changes, though both transcript and protein levels were mildly correlated (*r* of 0.503 and 0.576 for PFKP and PKM, respectively). These results indicate increased glycolytic flux in ccRCC consistent with the Warburg effect.

### Upstream target activation and inhibition

**Fig 5:**
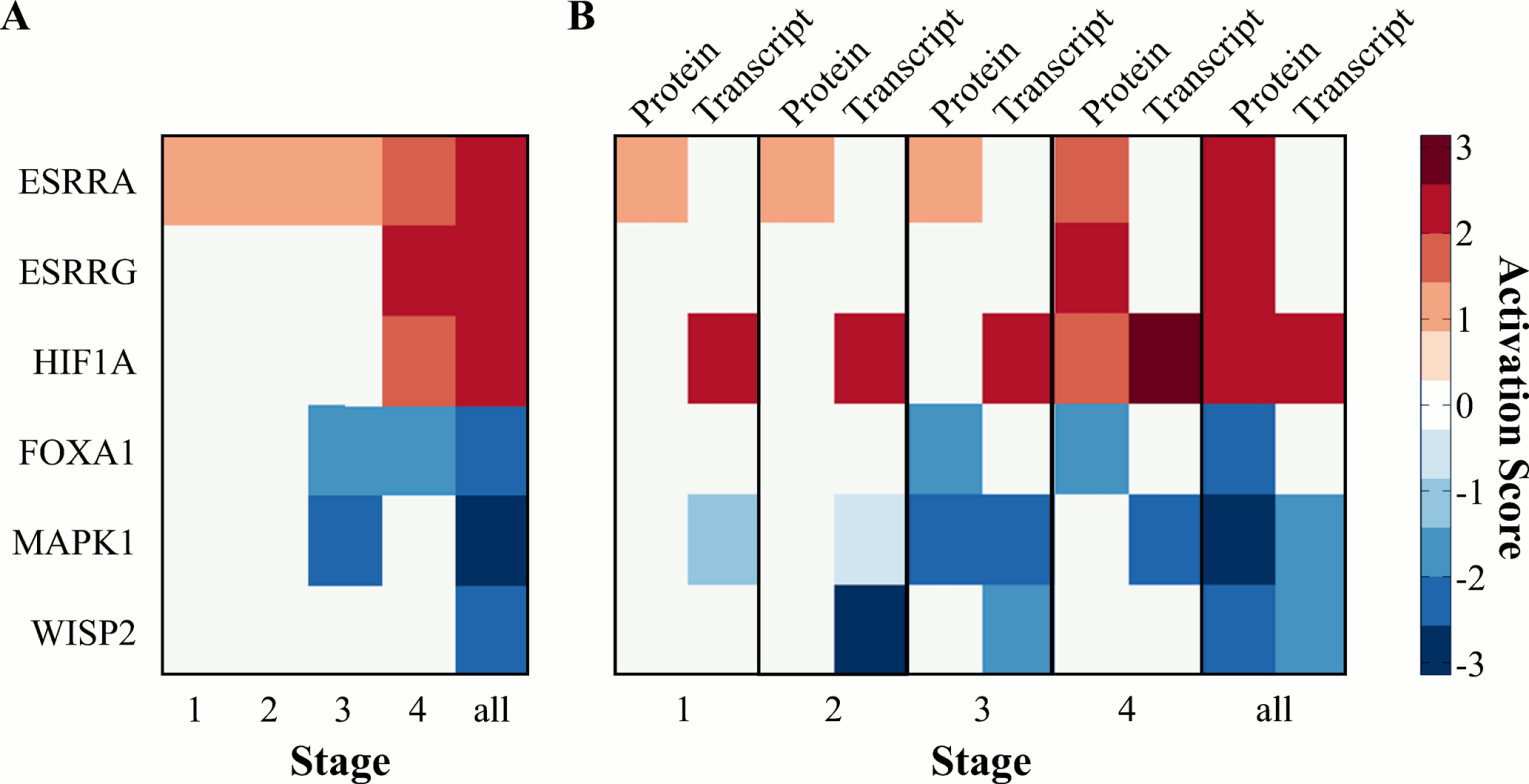
Upstream regulation with stage. (A) Heatmap of six upstream targets predicted to be activated/inhibited in tumor versus normal at each ccRCC stage and using all stage data together. Scale is activation z-score, with ≥ 2 being likely activation and ≤ -2 being likely inhibition. Using all stage data together, activated or inhibited upstream targets (ESRRA, ESRRG, HIF1A, FOXA1, MAPK1, and WISP2) are shown. (B) Heatmap of six upstream targets predicted to be activated/inhibited in tumor versus normal-adjacent tissues using the proteomic data set. Corresponding activation z-scores from transcriptomic data analysis are included to demonstrate conserved trends at each ccRCC stage and using all stage data together.

Using fold-change data for the 344 differentially abundant proteins, upstream regulator analysis was performed with IPA using data for each stage, as well as all 84 pairs, to predict activation or inhibition of regulators based on changes in protein abundance. There were six upstream targets predicted to be activated or inhibited in tumor versus normal-adjacent samples (Fig 5A). Of these, three were likely activated (activation z-score ≥ 2), estrogen-related receptor-α (ESRRA), ESRR-γ (ESRRG), and HIF1A, and three were likely inhibited (activation z-score ≤ -2), WNT1 inducible signaling pathway protein 2 (WISP2; also referred to as CCN5), FOXA1, and MAPK1. Except for ESRRG and MAPK1, the activation score was only significant when the complete data set was used (*i*.*e*., “all”). To further evaluate the predicted upstream changes, the downstream stage specific protein abundance changes associated with HIF1A, ESRRA, ESRRG, WISP2, FOXA1, and MAPK1 were plotted (S5 Fig). The activated networks (HIF1A, ESRRA, ESRRG) had many proteins that were increased in tumor tissues that overlapped between the three targets, such as ENO1/ENO2, ALDOA, and LDHA.

Next, the transcriptomic results were subjected to the same analysis in order to evaluate whether the proteomic based activation/inhibition predictions were supported at the transcript level. Only data for the six upstream targets identified with the proteomic dataset were compared to the activation z-scores calculated using the proteomic data (Fig 5B). Of the six upstream targets found to be activated/inhibited based on the proteomic data, only HIF1A, WISP2, and MAPK1 were supported by the analysis based on transcriptomic data (the other three upstream targets were absent from the transcriptomic based upstream regulator analysis results). Interestingly, for all three of these targets, activation/inhibition is predicted by the transcript data at an earlier ccRCC stage when using transcriptomic data. Specifically, HIF1A is predicted to be activated at all stages using transcript data, while using protein data, HIF1A is only predicted to be activated when all the data is used. Similarly, WISP2 is predicted to be inhibited at stage II and III when using the transcriptomic data, while only the full protein data set predicted it to be inhibited. On the other hand, MAPK1 is predicted to be inhibited at stage III and IV based on the transcript data, which aligns well with predictions based on the protein data. The agreement between the two analyses provided increased confidence in the proteomic results and highlights potential therapeutic targets of ccRCC.

### Candidate markers of aggressive ccRCC

**Fig 6:**
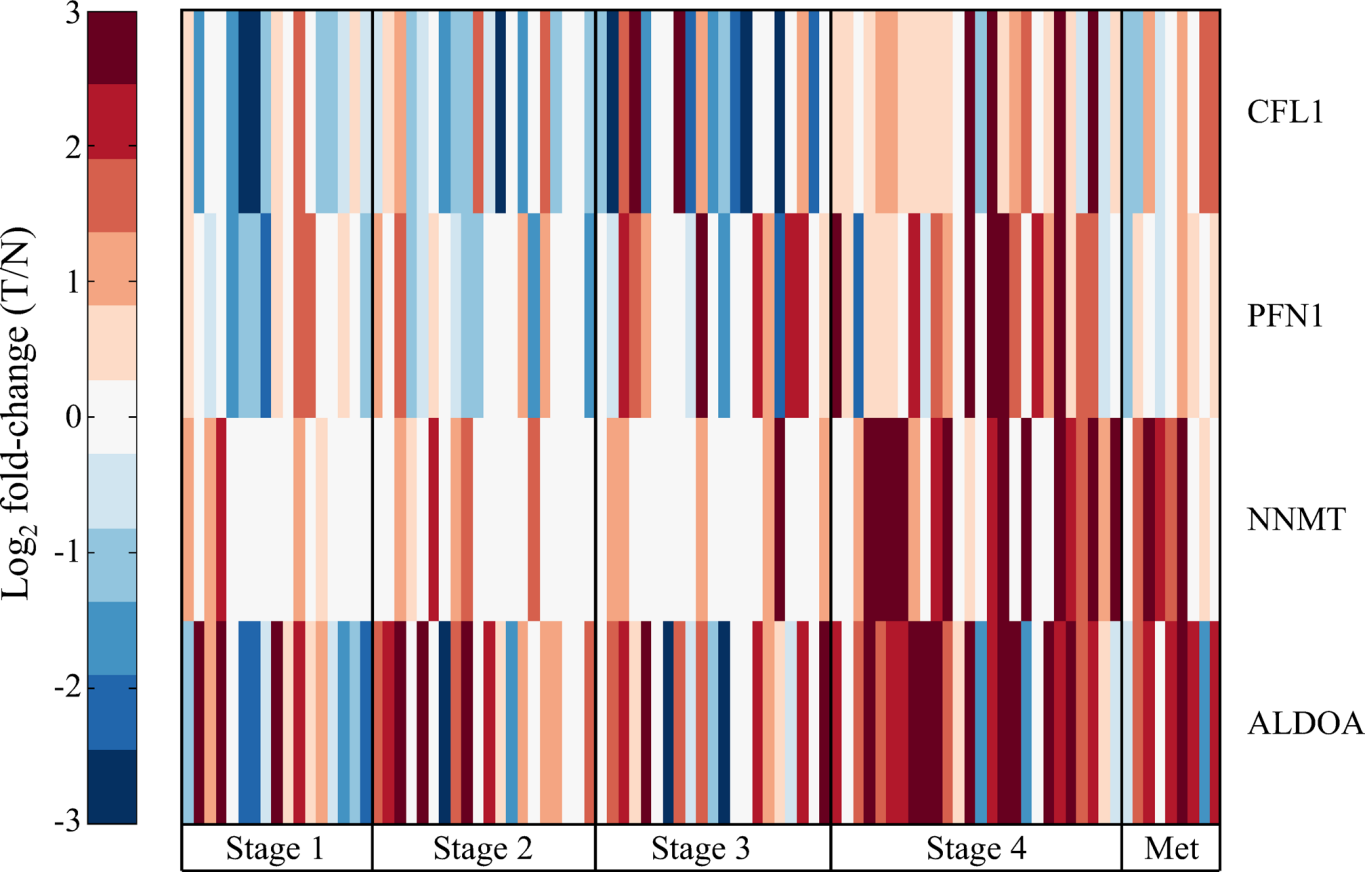
Candidate markers of advanced stage ccRCC. Heatmap of log_2_ fold-change in protein abundance of four candidate markers of late stage ccRCC, cofilin-1 (CFL1), profilin-1 (PFN1), nicotinamide N-methyltransferase (NNMT), and fructose-bisphosphate aldolase A (ALDOA), in paired tumor and normal-adjacent tissues from 84 individuals, as well as 9 pairs that also included metastasis tissue.

In addition to evaluating the proteomic and transcriptomic data to better understand the systematic molecular changes in ccRCC, we were also interested in whether there were proteins that could be used to discriminate stage IV ccRCC specifically. Of the 344 differentially abundant proteins, 50 were significantly different at stage IV only. To evaluate the discriminatory power of these 50 proteins, receiver operator characteristic (ROC) curves were constructed and area under the curve was estimated (AuROC). Of the 50 proteins, four had an AuROC > 0.7: cofilin-1 (CFL1), profilin-1 (PFN1), nicotinamide N-methyltransferase (NNMT), and fructose-bisphosphate aldolase A (ALDOA). All four proteins were increased at stage IV (Fig 6), yet CFL1, PFN1, and ALDOA were decreased at early stages relative to normal tissue. In the case of NNMT and ALDOA these trends were also reflected in metastasis tissues, while CFL1 and PFN1 levels were only slightly increased in metastasis tissues. These results highlight the dynamic nature of the molecular progression of ccRCC in addition to identifying candidate markers of ccRCC aggressiveness.

## Discussion

We present here the results of a comprehensive proteomic analysis of ccRCC tissues grouped by histopathology stages along with proteotranscriptomic analysis using previously reported gene expression array data from the same tissues. The goal of this analysis was not only to catalog proteins responsible for the molecular phenotype of ccRCC, but also evaluate stage dependent changes that reflect the molecular pathogenesis of ccRCC progression. These results confirm previous studies characterizing metabolic dysfunction in ccRCC [19, 21, 22, 24, 27, 28, 35], while also highlighting upstream gene targets that are predicted to be activated/inhibited using multi-domain analysis. Utilizing data across heterogeneous biological domains of the same samples not only strengthened these conclusions but also demonstrated the complementary nature of such an approach. Additionally, general ccRCC candidate biomarkers were observed, as well as stage specific markers related to high mortality metastatic ccRCC. These results highlight the benefits of a combined proteotranscriptomic approach and lay the foundation for future studies to confirm candidate therapeutic targets.

A hallmark feature of ccRCC is that cells undergo a metabolic shift consistent with the Warburg effect. These changes have been confirmed at the gene transcript level in ccRCC [19], and recently a series of proteomic analyses have confirmed similar widespread effects at the protein level [22–25, 35]. These studies identified between 770 and 1900 proteins with 30 to 350 differentially abundant between normal and tumor ccRCC tissues. Similarly, in the current study using a pooled sample approach we identified 1551 proteins, 290 of which were differentially abundant, while using individual samples we identified 783 proteins, 344 of which were differentially abundant. Using pathway enrichment analysis metabolic dysfunction was evident in ccRCC at all stages, as well as pathways involved in biosynthesis. Using data from recent proteomic studies of ccRCC [22, 24, 25, 35], similar pathway enrichment patterns were observed (S5 Table). In the current study we found that the key glycolytic enzyme PFKP was increased in all stages of ccRCC by both proteomic analyses and IHC. The companion transcriptomic analysis and prior proteomic studies [24, 25] also identified PFKP as being increased in ccRCC. This protein regulates an important control point in glycolysis and is an essential enzyme to drive glycolysis despite downstream feedback (as reviewed by [14]). Additionally, we observed increased PKM at all stages by both proteomic analyses and IHC, similar to other studies [22, 24, 25, 28, 35], which is another key change driving pyruvate production, highlighting potential roles PKM can play in tumor progression (as reviewed by [36]). In the current study we also observed decreased abundance of TCA cycle proteins, similar to results at the transcript level [19], specifically MDH2 and ACO2, which is similar to other proteomic studies [22, 24, 25, 35]. A recent metabolomic study found increased levels of citrate, *cis*-aconitate and succinate in ccRCC [27]. This could be partly explained by significantly decreased ACO2 protein abundance, but highlights that discrepancies between metabolite and proteotranscriptomic or proteomic and transcriptomic results should be investigated by acquiring data across domains in parallel on the same sample set. Overall, these results provide detailed empirical support for metabolic dysregulation and the Warburg effect in ccRCC and will help to improve our understanding of the underlying metabolic changes associated with ccRCC.

Relative to other cancers, VHL inactivation is relatively specific to ccRCC and is both critical to ccRCC evolution [37] and common across ccRCC [6, 7]. Inactivation of VHL leads to the stabilization of HIF1A and HIF2A (also referred to as EPAS1) [8, 9], with the former being responsible for many downstream changes related to the Warburg effect [13]. In the current study, HIF1A activation was evident by proteomic analysis of all tissue samples, but not within stages. In contrast to this, the companion transcriptomic data indicated increasing HIF1A activation with ccRCC stage, though HIF1A mRNA levels were significantly decreased 1.25-fold in tumor tissue. Alternatively, in the transcriptomic data HIF2A expression was significantly increased 1.4-fold and was predicted to be activated in stage IV ccRCC samples (activation scores increased with stage from 1.3 at stage I to 2.2 at stage IV). This apparent HIF2A activation is supported by the analysis of the proteomic data that indicated activation of ESRRA and ESRRG. Interestingly, although there is relatively low overlap between differentially abundant proteins detected in the current study and previous proteomic analyses [22, 24, 25, 35] (7 to 38%; S6 Fig), upstream analysis of these studies indicates ESRRA and ESRRG activation (S6 Table). Both of these nuclear receptors have increased expression in breast cancer [38] and ovarian cancer [39], while in neuroblastoma ESRRA expression has been shown to increase HIF2A expression and correlates with poor survival [40]. Given the additional involvement of ESRRA and ESRRG predicted by the proteomic data, HIF2A likely plays a key role in ccRCC, especially in later stages. A survey of 160 ccRCC tumors found that VHL-deficient tumors expressed either HIF1A and HIF2A or just HIF2A, and that tumors expressing only HIF2A had higher c-Myc activity and higher stage [9], correlating with the known proliferative effects of c-Myc in ccRCC [41]. Clearly the VHL/HIF1A/HIF2A axis is important in ccRCC development and proliferation, and these results highlight the potential involvement of ESRRA and ESRRG in driving these changes.

In addition to identifying upstream targets related to HIF1A and HIF2A, the proteomic data analysis predicted WISP2 (also referred to as CCN5) inhibition in ccRCC tissues. Using the transcriptomic data, WISP2 was predicted to be activated at stage II, and was significantly increased at the transcript level at stage II and III (1.5- and 2.7-fold, respectively). At the protein level, WISP2 activation was supported by increased vimentin and fibronectin levels, which have been previously reported in ccRCC [21, 25, 42, 43] (S6 Table), and decreased keratin 8 levels. In gastric [44], breast [45] and pancreatic cancer [46], WISP2 is a known tumor suppressor and likely regulates invasion and motility through MMPs [47]. The involvement of WISP-2 in ccRCC has not been reported,and additional targeted experiments should be performed to confirm its possible role in ccRCC progression. Overall, these results highlight the complementary nature of proteomic and transcriptomic analyses when used in conjunction to identify and confirm molecular changes.

To date there have been numerous genomic, transcriptomic, and proteomic analyses of ccRCC tissue, but there has not been a study that interrogates multiple biological domains of the same samples. In a prior study, similar ccRCC tissues were analyzed by cDNA arrays and 2D-GE, identifying 119 and 334 significantly different genes and proteins, respectively, with an overlap of only 12 genes/proteins [48]. Companion proteomic studies to the seminal TCGA studies are being published, such as the follow up proteomic analysis of colorectal cancer by Zhang *et al*. [26]. Similar to the analysis presented in the current study, they evaluated the correlation of mRNA levels and protein abundance and found the mean Spearman’s rank correlation coefficient of 0.23, which is similar to our results (average *r* = 0.157) and other similar study designs [49, 50]. This low level of correlation is not entirely surprising since it is understood that the transcriptome and proteome are not linearly related [49]. For example, in the Zhang *et al*. proteomic study only 60% of a small group genes of interest identified at the mRNA level were also significant at the protein level. In the current study we utilized the transcriptomic data to support changes seen in the proteomic data, but, as is evident with the ESRRA and ESRRG findings (*i*.*e*., activation is not predicted using transcriptome data), changes at the protein level may not be directly mirrored at the transcriptome level. Protein changes are likely more indicative of broader phenotypic changes, and also likely represent a non-linear composite effect of many upstream message level changes. Using data from both biological domains of the same samples has improved the confidence of our findings and may serve to limit false discoveries in future confirmation experiments.

Early diagnosis of ccRCC significantly improves patient outcomes, and for this reason there have been numerous studies looking for transcriptomic, peptidomic, proteomic, miRNA, and metabolomic signatures of late stage ccRCC in tissue, serum, and urine (reviewed by [51–53]). To date there are no confirmed biomarkers that can be used for screening late-stage ccRCC, though there are many candidate markers of ccRCC relative to normal tissue. Many of these markers were detected in the current study, such as MnSOD [24, 54] and vimentin [55] (to name a few), but our focus was to investigate candidate markers specific to late-stage ccRCC. After eliminating non-stage specific markers of ccRCC, four proteins were identified that were specific to stage IV. Three of these proteins have been confirmed by western blotting and/or IHC in ccRCC. Cofilin (CFL1) has been confirmed to be increased in ccRCC [54] but has not been confirmed in late-stage ccRCC, though it is known to be associated with metastasis in many solid tumors [56–58]. Profilin (PFN1) has been shown to be increased in metastatic ccRCC by IHC [35] and is also a candidate marker of bladder cancer metastasis [59], though it is also down-regulated in numerous other cancers (as discussed by [35]). Nicotinamide N-methyltransferase (NNMT) has recently been shown to be an interesting candidate marker of aggressive ccRCC by two recent studies: Lebdai *et al*. demonstrated NNMT overexpression by western blotting in ccRCC tissues with high SSIGN scores [23], while Zaravinos *et al*. identified NNMT following a large meta-analysis of five published transcriptomic data sets and confirmed overexpression by IHC in ccRCC tissues [60]. Our data also indicate fructose-bisphosphate aldolase A (ALDOA) is a candidate marker of late-stage ccRCC, which has been observed in other ccRCC studies, but not specifically as a marker of late-stage ccRCC. ALDOA has been identified as a marker of late-stage colorectal cancer [61] and lung squamous cell carcinoma [62], and is involved in osteosarcoma metastasis [63]. Further studies are required to confirm that increased abundance of these four proteins correlate specifically to late-stage ccRCC.

One of the most defining traits of ccRCC is late-stage tumor diversity [37] along with intratumor heterogeneity [64]. Developing molecular tools to subtype ccRCC beyond TNM staging are key, but also developing ways to accurately evaluate a heterogeneous genomic, and therefore molecular, landscape are key. Based on the results herein, it is evident that there are many shared traits among ccRCC stages related to the Warburg effect, but it seems likely that there are major differences between tumor stage related to the VHL/HIF1A/HIF2A axis that likely affect tumor aggressiveness and treatment. Developing study designs that focus on stratified samples (by *VHL* and/or HIF1A/HIF2A status) along with multiple samples from tumors and a multi-domain approach (such as proteotranscriptomic) is necessary to more clearly define the molecular pathogenesis of ccRCC and identify new therapeutic targets that are required for metastatic ccRCC.

## Supporting Information

S1 Fig Positive and negative controls of immunohistochemical staining. (A-C) Controls for 6-phosphofructokinase (PFKP antibody): (A) Negative control on breast cancer tissue. (B) Positive control at 1:1000 and (C) 1:2000 on breast cancer tissue. (D-F) Controls for and pyruvate kinase (PKM2 antibody): (D) Negative control on normal lung tissue. (E) Positive control at 1:2500 and (F) 1:3000 on lung cancer tissue. Scale bar is 200 μm.
(TIF)

S2 Fig Replicate variability within pooled analysis. Variability between biological replicates in the pooled analysis was visualized using scatter plots of adjusted spectral counts for the 1551 proteins identified, and the Pearson's linear correlation coefficient (*r*) was calculated for each pair. There from four stage I tumor (t1_#), two stage I normal-adjacent (n1_#), two stage II tumor (t2_#), two stage II normal-adjacent (n2_#), two stage III tumor (t3_#), two stage III normal-adjacent (n3_#), two stage IV tumor (t4_#) and two stage IV normal-adjacent (n4_#) pooled samples. The axis identifiers are the same used in S2 Table. The average *r* within replicates was 0.8835.
(TIF)

S3 Fig Comparison of proteomic results from pooled and individual samples. (A) Overlap between the proteins identified in the pooled and individual sample analysis. (B) Comparison of directionality agreement between the 663 overlapping proteins using log_2_ fold-change (tumor/normal-adjacent). There were 123 proteins changing in different directions but these differences were not dramatic. (C) Overlap of differentially abundant proteins identified using each approach.
(TIF)

S4 Fig Distribution of mRNA expression levels across all stages. Data from 94 arrays with 54,677 probes was RMA normalized and evaluated using a moderated *t*-test comparing all tumor normal-adjacent pairs. RMA normalized expression values were plotted against Benjamini-Hockberg (BH) adjusted *p*-values. Emphasized in red are probes with BH adjusted *p* < 0.001 and absolute fold-change ≥ 4 (1003 genes).
(TIF)

S5 Fig Predicted upstream effects. Using protein abundance data from all four stages, HIF1A, ESRRG, and ESRRA were predicted to be activated and WISP2, FOXA1, and MAPK1 were predicted to be inhibited. Bar graphs are four stage and all stage comparisons. Image generated with QIAGEN’s Ingenuity® Pathway Analysis.
(TIF)

S6 Fig Similarity of other shotgun proteomics studies with the current proteotranscriptomic study. Differentially abundant proteins and genes from this study (pooled, individual and 94 array) were compared to differentially abundant proteins identified in four other studies (granular proteomic results from Lebdai *et al*.[23] were not available): Perroud *et al*.[25] Supp. Table 3 (180 proteins), Masui *et al*.[35] Supp. Table 5 (29 proteins), White *et al*.[22] Supp. Table 7 (55 proteins), and Zhao *et al*.[24] Online resource 1 (213 proteins). Two separate list of unique gene symbols for proteins or genes that increased (n = 458) or decreased (n = 719) in ccRCC tissue across data sets were created, and a binary matrix of presence/absence was constructed for these across experiments. A dendrogram for each list was created using the unweighted pair group method with arithmetic mean method (euclidian distance; Matlab). Specifically, of the increasing proteins the overlap of each study with the individual analysis presented herein was 28.4%, 10.5%, 12.6%, and 21.1% for Perroud, Masui, White, and Zhao respectively, and of the decreasing proteins the overlap of each study with the individual analysis was 35.7%, 7.0%, 8.2%, and 37.7% respectively. The overlap between the individual and pooled analysis described in S3 Fig is due to the 59% overlap in decreasing proteins resulting in their proximity on the dendrogram.
(TIF)

S1 Table Data organization key. The tissues analyzed in this study were also part of two previous expression array studies. The proteomic data has been deposited to the ProteomeXchange Consortium via the PRIDE partner repository with the dataset identifier PXD003271 and 10.6019/PXD003271. The PRIDE repository assigns assay numbers, but maintains mzIdentML filenames (.mzid) for each run. These correspond directly to the array data that was deposited on the NCBI Gene Expression Omnibus (GSE 53757). These.cel files have been linked to the respective proteomic mzIdentML files.
(XLSX)

S2 Table Proteomic analysis of pooled samples. Proteomic data from the pooled samples were evaluated by using the asp values for each protein to perform a Wilcoxon rank sum test using the exact method followed by a Benjamini-Hochberg (BH) procedure to correct for multiple hypothesis testing. Whether the sample is from pooled tumor tissues or normal-adjacent is indicated by a 't' or 'n', respectively, followed immediately by a number which refers to stage. Fold-change (FC) values were determined by taking the log2 of each spectral count (in case of zero, 0.5 was used), and log2 fold-change was the difference in averages.
(XLSX)

S3 Table Proteomic analysis of individual samples. Normalized spectral count values for the 783 proteins identified across 177 samples is given along with sample information and protein information.
(XLSX)

S4 Table Statistical analysis of fold-change protein differences between tumor and normal-adjacent pairs. For each of the 783 proteins identified in the individual analysis log_2_ fold-change is given as well as *p*-value (moderated *t*-test) and BH adjusted *p*-value for each stage and using all samples.
(XLSX)

S5 Table Pathway enrichment analysis of previously published proteomic analyses of ccRCC. Differentially abundant proteins from this study (pooled and individual analyses) were compared to differentially abundant proteins identified in four other studies (granular proteomic results from Lebdai *et al*.[23] were not available): Perroud *et al*.[25] Supp. Table 3 (180 proteins), Masui *et al*.[35] Supp. Table 5 (29 proteins), White *et al*.[22] Supp. Table 7 (55 proteins), and Zhao *et al*.[24] Online resource 1 (213 proteins). Pathway enrichment analysis was performed using IPA as described in the current study and the top 15 pathways with more than one molecule and *p* < 0.05 are given (ranked by *p*-value).
(XLSX)

S6 Table Upstream regulator analysis of previously published proteomic analyses of ccRCC. Differentially abundant proteins from this study (pooled and individual analyses) were compared to differentially abundant proteins identified in four other studies (granular proteomic results from Lebdai *et al*.[23] were not available): Perroud *et al*.[25] Supp. Table 3 (180 proteins), Masui *et al*.[35] Supp. Table 5 (29 proteins), White *et al*.[22] Supp. Table 7 (55 proteins), and Zhao *et al*.[24] Online resource 1 (213 proteins). Upstream regulator analysis was performed using IPA as described in the current study and the top 15 targets are given (ranked by *p*-value). The activation z-score is given and an activation z-score ≥ 2 is considered activated and ≤ -2 is considered inhibited. The six targets identified in the current study as being activated or inhibited (ESRRA, ESRRG, HIF1A, FOXA1, MAPK1, and WISP2; Fig 5) are highlighted in bold or listed in bolded italics below the top 15 targets if not included in the top 15 (ranked by *p*-value). Also, EPAS1 (also refered to as HIF2A) is listed if present since its activation is implied by ESRRA/ESRRG activation and the transcriptomic analysis in the current study.
(XLSX)
