# Supplementary figures and images for: Proteotranscriptomic Analysis Reveals Stage Specific Changes in the Molecular Landscape of Clear-Cell Renal Cell Carcinoma
Source: PLoS One. 2016 Apr 29;11(4):e0154074. doi: 10.1371/journal.pone.0154074 (PMC4851420; doi:10.1371/journal.pone.0154074)

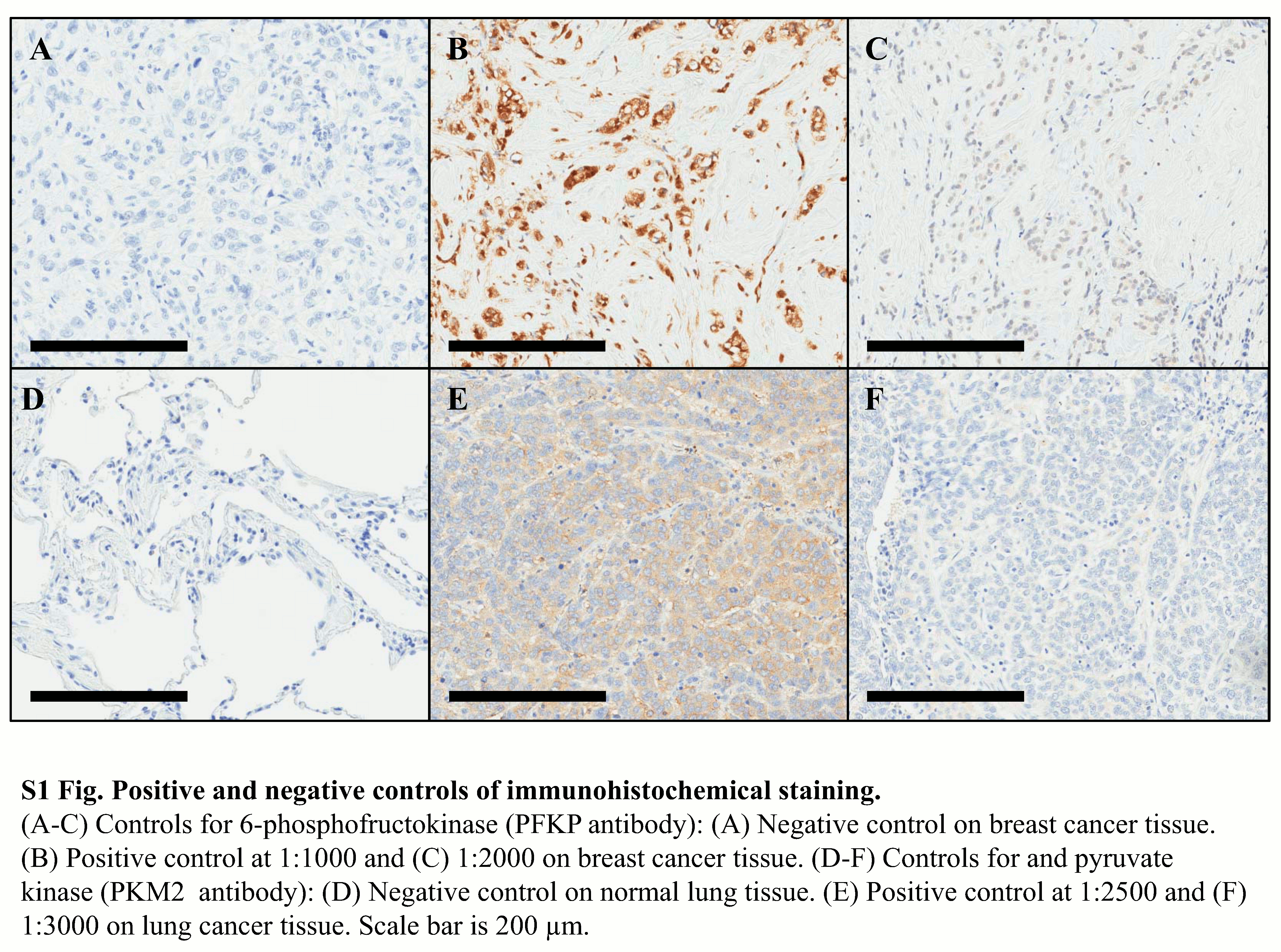

Supplement: S1 Fig — (A-C) Controls for 6-phosphofructokinase (PFKP antibody): (A) Negative control on breast cancer tissue. (B) Positive control at 1:1000 and (C) 1:2000 on breast cancer tissue. (D-F) Controls for and pyruvate kinase (PKM2 antibody): (D) Negative control on normal lung tissue. (E) Positive control at 1:2500 and (F) 1:3000 on lung cancer tissue. Scale bar is 200 μm. (TIF) [file pone.0154074.s001.tif]

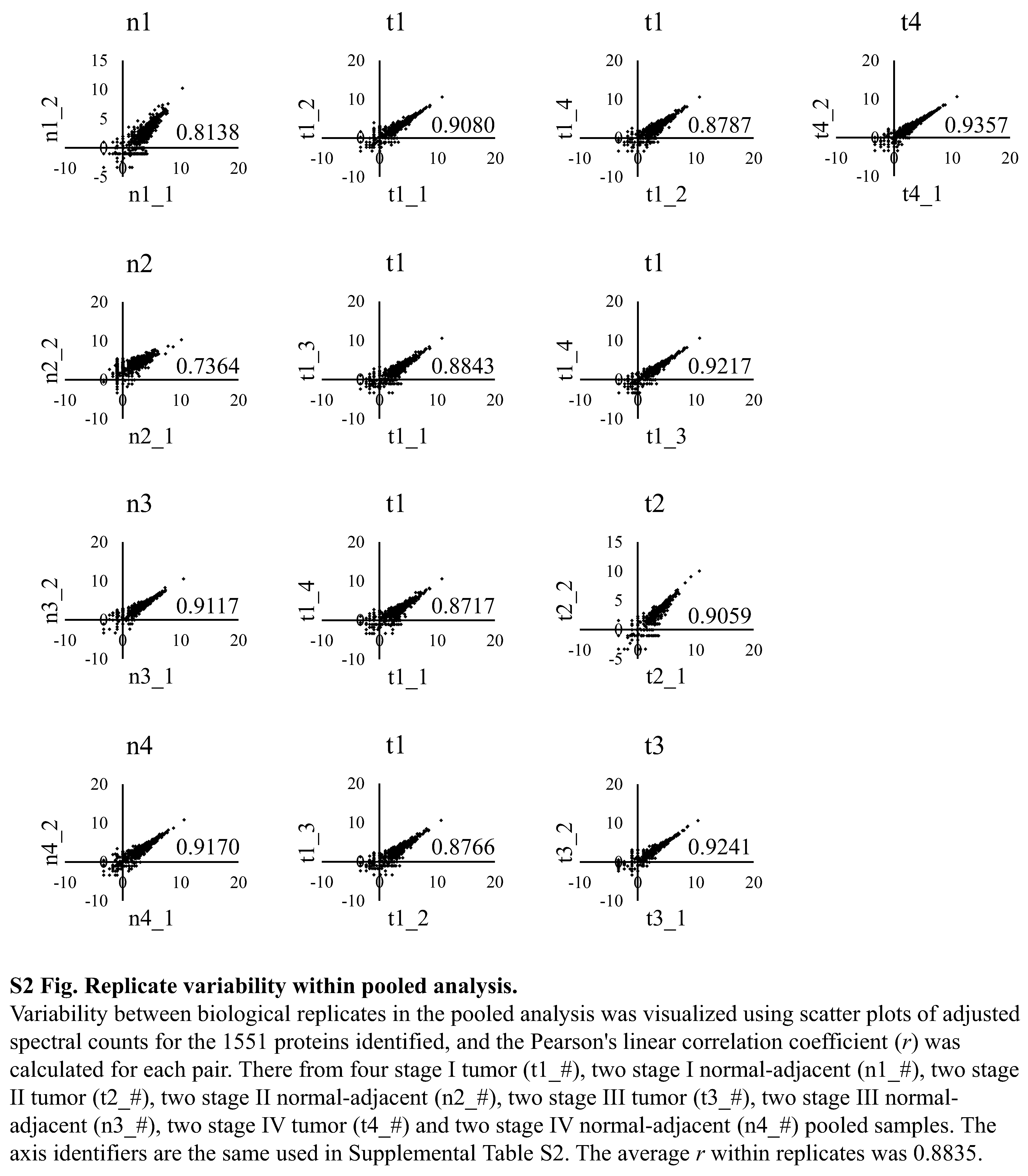

Supplement: S2 Fig — Variability between biological replicates in the pooled analysis was visualized using scatter plots of adjusted spectral counts for the 1551 proteins identified, and the Pearson's linear correlation coefficient (r) was calculated for each pair. There from four stage I tumor (t1_#), two stage I normal-adjacent (n1_#), two stage II tumor (t2_#), two stage II normal-adjacent (n2_#), two stage III tumor (t3_#), two stage III normal-adjacent (n3_#), two stage IV tumor (t4_#) and two stage IV normal-adjacent (n4_#) pooled samples. The axis identifiers are the same used in S2 Table. The average r within replicates was 0.8835. (TIF) [file pone.0154074.s002.tif]

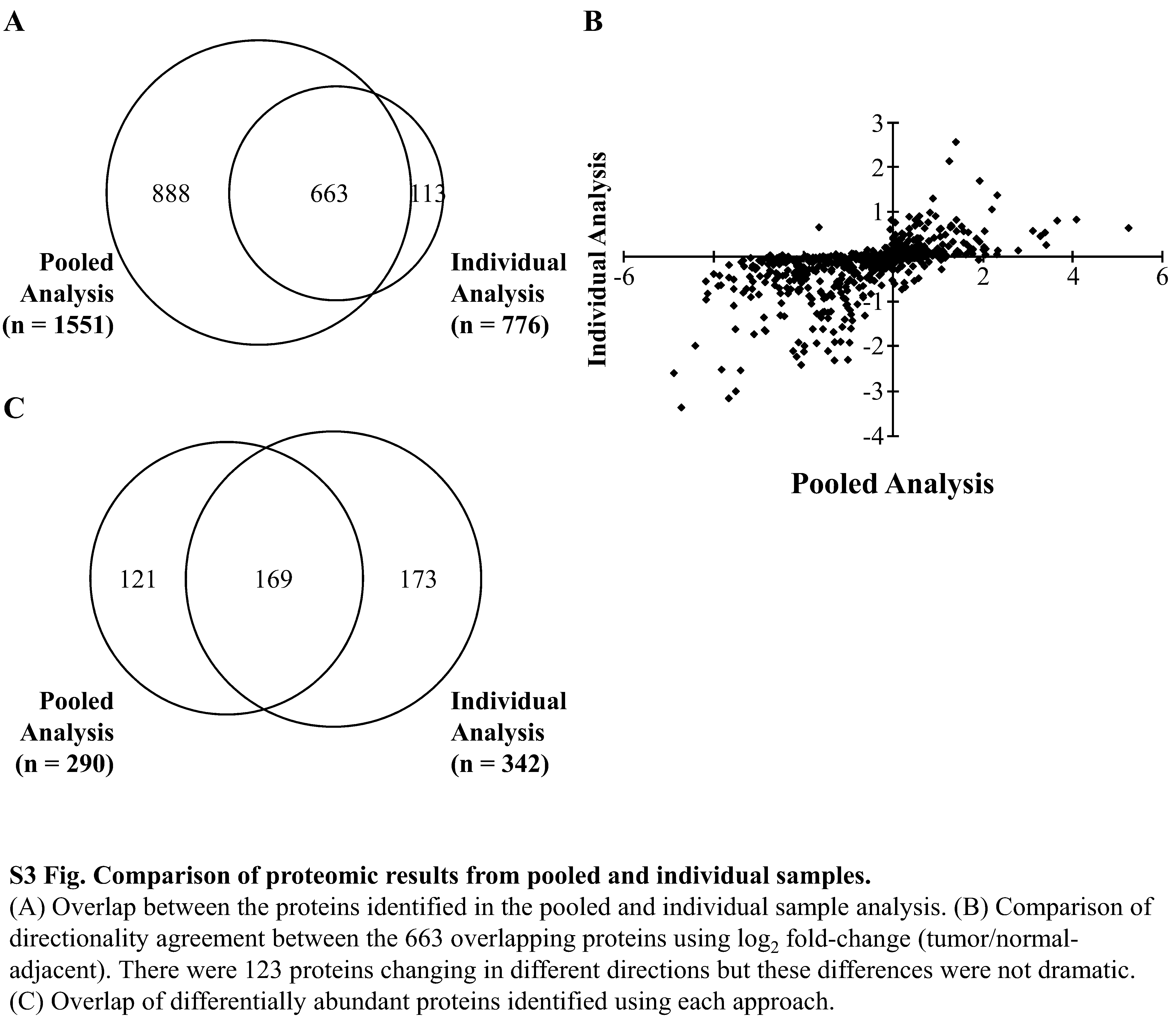

Supplement: S3 Fig — (A) Overlap between the proteins identified in the pooled and individual sample analysis. (B) Comparison of directionality agreement between the 663 overlapping proteins using log2 fold-change (tumor/normal-adjacent). There were 123 proteins changing in different directions but these differences were not dramatic. (C) Overlap of differentially abundant proteins identified using each approach. (TIF) [file pone.0154074.s003.tif]

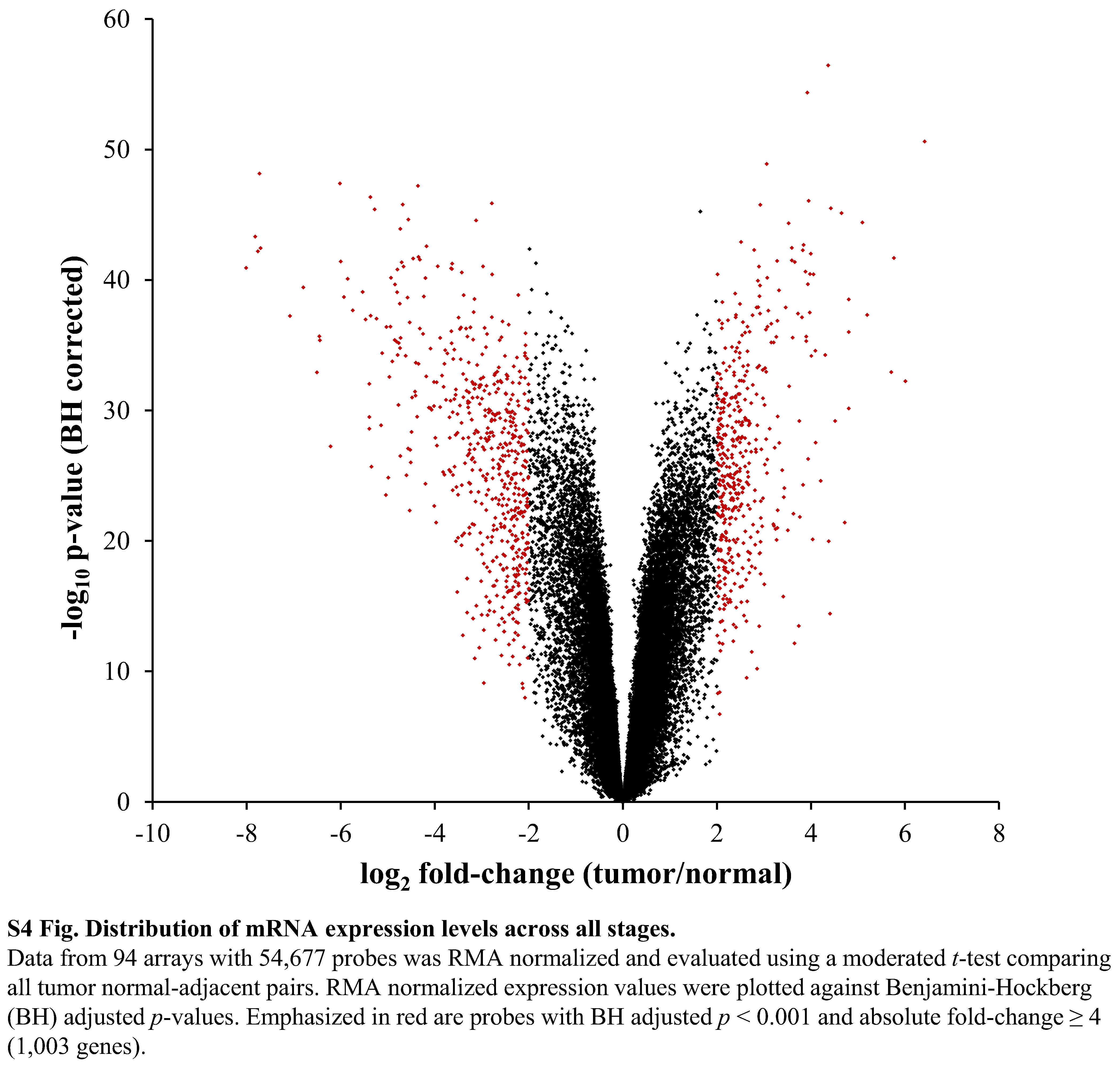

Supplement: S4 Fig — Data from 94 arrays with 54,677 probes was RMA normalized and evaluated using a moderated t-test comparing all tumor normal-adjacent pairs. RMA normalized expression values were plotted against Benjamini-Hockberg (BH) adjusted p-values. Emphasized in red are probes with BH adjusted p < 0.001 and absolute fold-change ≥ 4 (1003 genes). (TIF) [file pone.0154074.s004.tif]

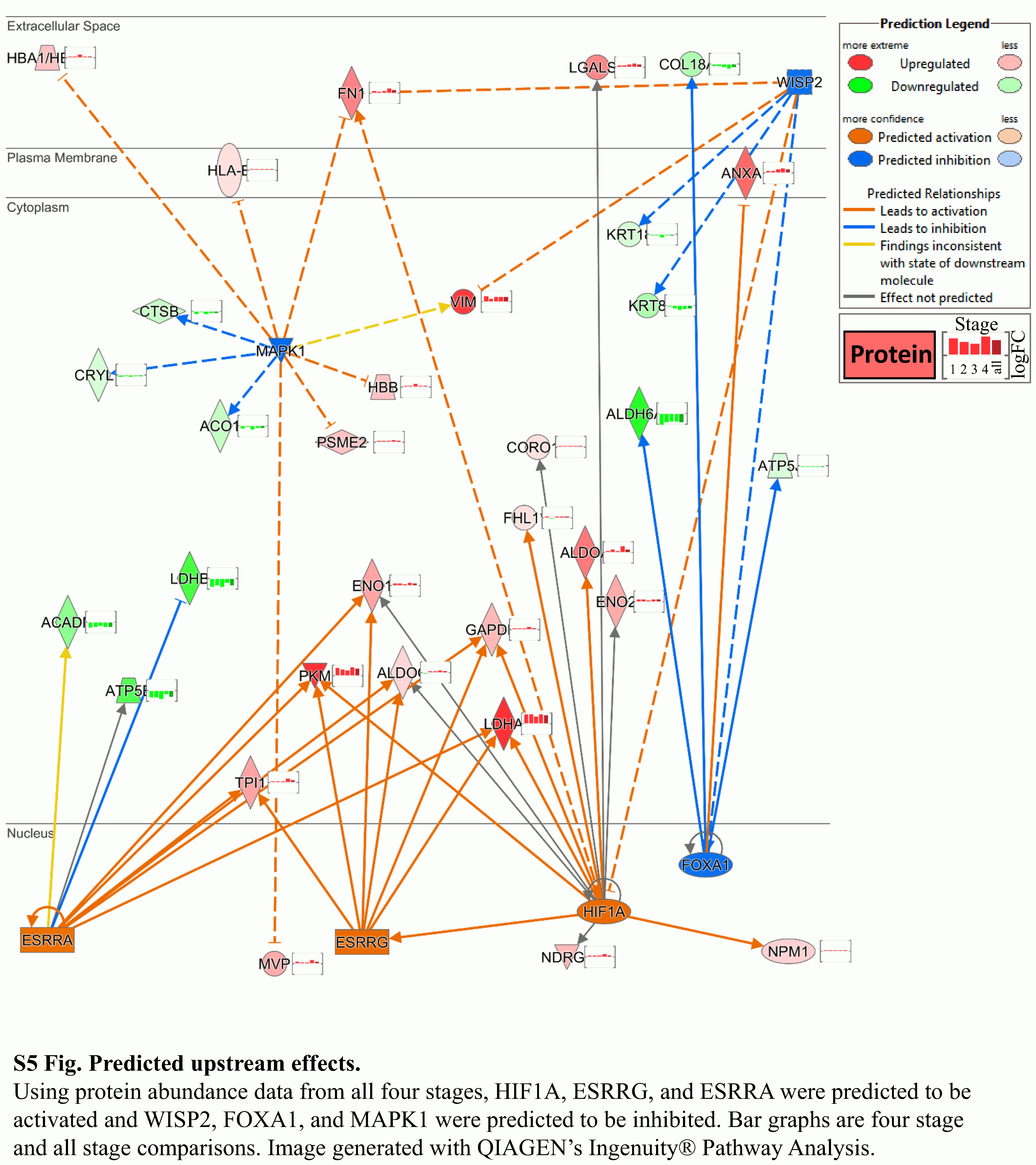

Supplement: S5 Fig — Using protein abundance data from all four stages, HIF1A, ESRRG, and ESRRA were predicted to be activated and WISP2, FOXA1, and MAPK1 were predicted to be inhibited. Bar graphs are four stage and all stage comparisons. Image generated with QIAGEN’s Ingenuity® Pathway Analysis. (TIF) [file pone.0154074.s005.tif]

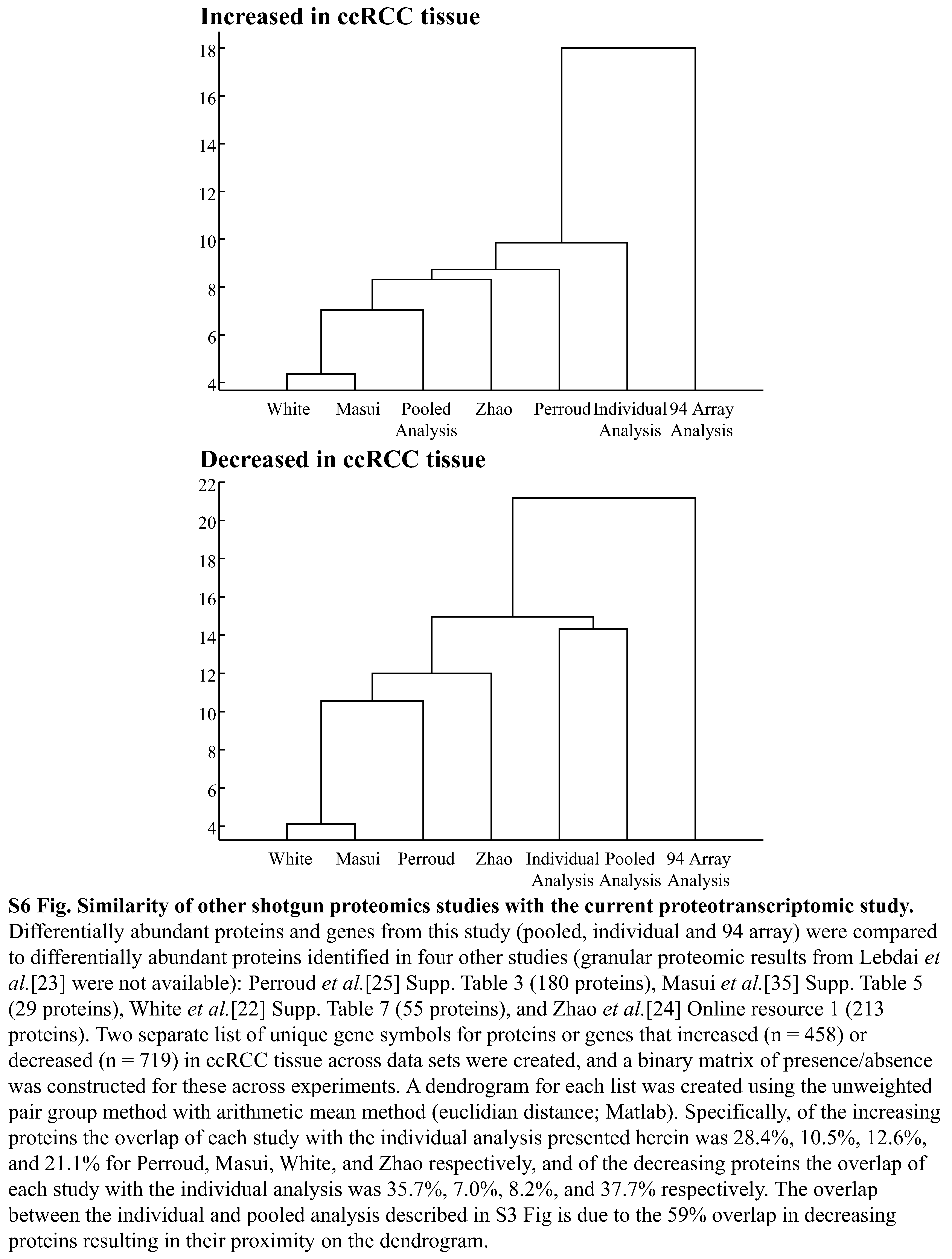

Supplement: S6 Fig — Differentially abundant proteins and genes from this study (pooled, individual and 94 array) were compared to differentially abundant proteins identified in four other studies (granular proteomic results from Lebdai et al.[23] were not available): Perroud et al.[25] Supp. Table 3 (180 proteins), Masui et al.[35] Supp. Table 5 (29 proteins), White et al.[22] Supp. Table 7 (55 proteins), and Zhao et al.[24] Online resource 1 (213 proteins). Two separate list of unique gene symbols for proteins or genes that increased (n = 458) or decreased (n = 719) in ccRCC tissue across data sets were created, and a binary matrix of presence/absence was constructed for these across experiments. A dendrogram for each list was created using the unweighted pair group method with arithmetic mean method (euclidian distance; Matlab). Specifically, of the increasing proteins the overlap of each study with the individual analysis presented herein was 28.4%, 10.5%, 12.6%, and 21.1% for Perroud, Masui, White, and Zhao respectively, and of the decreasing proteins the overlap of each study with the individual analysis was 35.7%, 7.0%, 8.2%, and 37.7% respectively. The overlap between the individual and pooled analysis described in S3 Fig is due to the 59% overlap in decreasing proteins resulting in their proximity on the dendrogram. (TIF) [file pone.0154074.s006.tif]
